# Hypoxia-Induced Matrix Metalloproteinase-13 Expression in Exosomes from Nasopharyngeal Carcinoma Enhances Metastases

**DOI:** 10.1038/s41419-018-0425-0

**Published:** 2018-03-07

**Authors:** Ying Shan, Bo You, Si Shi, Wei Shi, Zhenxin Zhang, Qicheng Zhang, Miao Gu, Jing Chen, Lili Bao, Dong Liu, Yiwen You

**Affiliations:** 1 Department of Otorhinolaryngology Head and Neck Surgery, Affiliated Hospital of Nantong University, Nantong, China; 2 Department of Neurosurgery, Affiliated Hospital of Nantong University, Nantong, China; 3 Co-innovation Center of Neuroregeneration, Jiangsu Key Laboratory of Neuroregeneration, Nantong University, Nantong, China

## Abstract

Exosomes are nano-vesicles secreted by tumor cells. Exosomes can transfer complex biological information and induce a diverse signaling response in a wide array of pathological conditions, such as hypoxia. Hypoxia is associated with aggressive phenotypes and poor outcomes in nasopharyngeal carcinoma (NPC) patients. Here, we analyzed the role of exosomes from hypoxic NPC cells in enhancing the metastases of normoxic cells in a hypoxia-induced factor-1α (HIF-1α)-dependent manner. HIF-1α rapidly accumulates and trans-activates hundreds of genes, such as matrix metalloproteinases (MMPs). We found that MMP-13 was over-expressed in exosomes and cells under hypoxic conditions. HIF-1α depletion in hypoxic CNE2 cells led to decreased MMP-13 levels in exosomes and significantly reduced cell migration and invasion. Moreover, exosomal MMP-13 significantly up-regulated Vimentin expression while decreasing E-cadherin levels in CNE2 cells in vitro and in vivo. Furthermore, MMP-13 levels were closely associated with HIF-1α expression (*r* = 0.679, *P* < 0.001), lymph node metastasis, clinical stage (all *P* < 0.05) and poor prognosis in NPC patients (*P* < 0.01). In conclusion, our findings suggest that the hypoxic exosomes were loaded with MMP-13, which could enhance migration and invasiveness and induce microenvironment changes to promote NPC aggressiveness.

## Introduction

Nasopharyngeal carcinoma (NPC) is a squamous epithelial cancer arising from the lateral wall surface of the nasopharynx^1,2^, and it has the highest metastatic potential among head and neck cancers. Distant metastasis is the major cause of treatment failure^3^. At the time of diagnosis, 74.5% of NPC patients present with regional lymph node metastasis, and 19.9% present with distant metastasis^3^. Post-treatment local relapse and particularly distant metastasis remain the major problematic issues that eventually lead to the death of patients with advanced NPC^4^. Further research of the molecular mechanisms of NPC tumorigenesis is urgently needed, especially regarding metastases, to improve the prognosis of patients.

It has become generally accepted that cancer progression is driven by hypoxic signaling, and the expression of hypoxia-related markers has been correlated with poor patient outcome in several tumor types, which could partly relate to tumor metastases^5^. Hypoxia is recognized as the most common features of the tumor microenvironment that contributes to tumor progression by activating adaptive transcriptional programs, thereby promoting tumor cell survival, motility, metastases and angiogenesis^6–8^. Clinical studies showed that 100% of primary NPC and 58% of cervical nodal metastases of NPC were found to contain hypoxic regions with over-expression of hypoxia-inducible factor-1α (HIF-1α) which is associated with an increased risk of metastases and patient mortality^4^. HIF-1α has become a prognostic factor as well as a potential therapeutic target of NPC^9^. HIF-1α can accumulate and rapidly trans-activate hundreds of target genes under hypoxia, such as angiogenic and proliferating factors, glucose transporters^10^ as well as matrix metalloproteinases (MMPs)^11^. MMP-13, a member of the MMP family, is often over-expressed in various tumors^12–16^, and it has been documented to increase the risk of metastases of cancers of the head and neck^14^ and melanoma^17^. The recent discovery of exosomes and their extracellular presence suggest a potential role of these regulatory molecules in defining the metastatic potential of cancer cells and mediating the microenvironment^16^. In previous work, we proved that exosomes from NPC containing MMP-13 which could mediate the tumor microenvironment, such as by facilitating tumor cell migration and invasion by inducing epithelial–mesenchymal transition (EMT)^18^.

Exosomes are nano-sized (50–100 nm in diameter) membrane-bound vesicles released by a variety of cell types, especially abundantly by tumor cells^19^. Recent evidence has highlighted a role for hypoxic tumor cell-derived exosomes in promoting tumor progression^20^. Exosomes may participate in intercellular signaling, e.g., via protein ligands in the exosome membrane that activate signaling receptors and downstream kinases, and through the transfer of protein, microRNAs (miRNAs), messenger RNAs (mRNAs) and signaling receptors to recipient cells^5^. Kucharzewska et al.^5^ found that exosomes constitute a potentially targetable mediator of hypoxia-driven tumor development and that the exosomal molecular signature could serve as a noninvasive biomarker to assess the oxygenation status and aggressiveness of malignant tumors. Given the observations that independently link the hypoxia microenvironment and exosome-mediated signals to invasive tumor phenotypes, it is of great interest to investigate whether hypoxia could promote tumor progression through altered exosomes release.

In the present study, we further investigated whether NPC cells exposed to hypoxia release exosomes containing a higher level of MMP-13 in an HIF-1α-dependent manner, therefore enhancing metastases by inducing EMT in vitro and in vivo. We found that over-expression of HIF-1α and MMP-13 could be involved in the carcinogenesis and development of NPC and that their over-expression was associated with patients’ poor prognosis. Taken together, MMP-13 over-expression was triggered by hypoxia/HIF-1α as an important mechanism to induced EMT and tumor invasion in NPC.

## Results

### Hypoxic nasopharyngeal cancer cell-derived exosomes induced metastases of normoxic cells

In previous work, we proved that MMP-13-containing exosomes from NPC could mediate the tumor microenvironment by facilitating tumor cell migration and invasion via the induction of EMT^18^. As hypoxia facilitates metastasis-associated processes, including the EMT, invasive migration and angiogenesis, it is of interest to investigate whether NPC cells could enhance the invasiveness and motility of naive NPC cells under hypoxic conditions by transferring information packaged in exosomes.

**Fig. 1:**
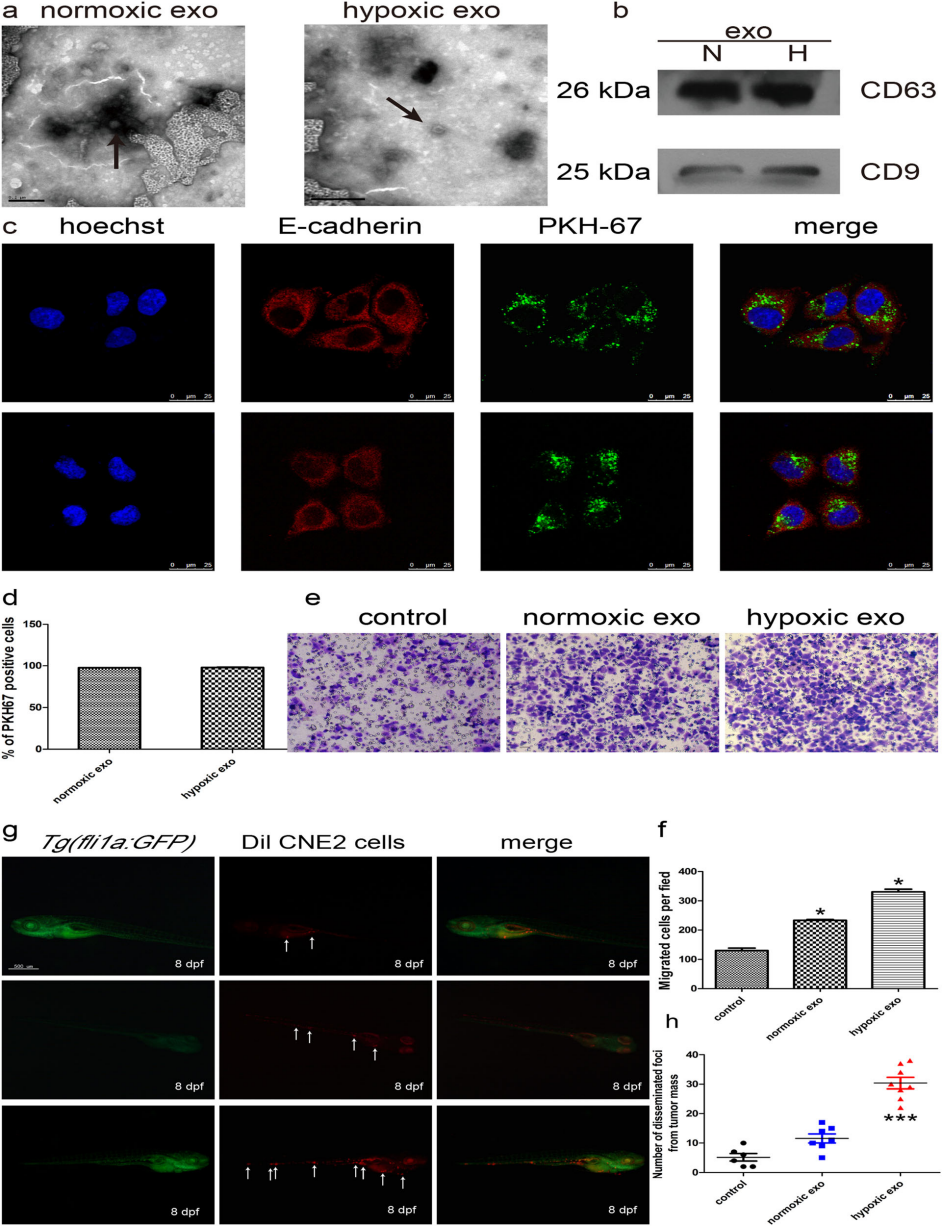
Hypoxic tumor-derived exosomes induced metastases of normoxic cells. **a** Electron micrograph of exosomes isolated from CNE2-CM. Scale bar, 200 nm. **b** Western blot analysis showing the presence of exosomal markers. **c** Confocal microscopy image showing the internalization of PKH67-labeled normoxic (upper) and hypoxic (lower) exosomes (green) by CNE2 cells. **d** Flow cytometry analyzed the PKH67-labeled exosomes uptaked by CNE2. **e**, **f** Analysis of tumor cell migration by transwell assay (**e**). The numbers of migratory cells were quantified (**f**). **g** Dil-labeled CNE2 cells were injected into the perivitelline space of 48 hpf (hours post fertilization) embryos and tumor cell invasion, dissemination and metastasis were detected using fluorescence microscopy at day 8 post injection. Arrows indicate tumor foci. Scale bar, 500 nm. Data represent at least three experiments performed in triplicate, * indicates 0.01 < *p* < 0.05; ***, *p* < 0.001

We incubated NPC cells under hypoxic conditions, and found that HIF-1α increased with a time dependency under oxygen deprivation conditions (Supplementary Figure 1a-f). We also observed morphological, protein and mRNA changes of EMT as well as the enhanced tumor migration in CNE2 cell lines under hypoxia (Supplementary Figure 2a-e). Exosomes were purified from the conditioned medium (CM) of normoxic and hypoxic CNE2 cells. Electron microscopy and immunoblot analysis confirmed the presence of exosomes (Fig. 1a, b). There was no significant difference between the morphology of exosomes under hypoxic conditions compared to the normal conditions.

Once secreted, exosomes deliver biologic information that is internalized by neighboring or distant cells. We sought to investigate whether exosomes derived from hypoxic tumor cells could affect normoxic cells. PKH67-labeled exosomes were incubated with normoxic CNE2 cells for 1 h. The recipient cells exhibited a high uptake efficiency, as indicated by fluorescence microscopy (Fig. 1c) and flow cytometry (Fig. 1d), without a significant difference between normoxic and hypoxic cell- derived exosomes (Fig. 1d). However, after 1 h of incubation with labeled exosomes, >90% of the recipient cells were positive for PKH67 fluorescence (Fig. 1c). Transwell assays showed that hypoxic exosomes could facilitate the metastasis capacity of normoxic CNE2 cells compared with normoxic exosomes (Fig. 1e, f). Recent studies show that zebrafish represents a promising alternative model in cancer research^21,22^. In these studies, Dil-labeled CNE2 cells mixed with the same amount of normoxic or hypoxic exosomes were injected at the blastula stage to explore the potential effect of exosomes in cancer metastases. At day 8 after implantation, CNE2 cells with hypoxic exosomes were significantly disseminated away from primary sites (Fig. 1g, h) compared with CNE2 cell with normoxic exosomes. These results suggested that hypoxic exosomes facilitated the metastasis of nasopharyngeal cancer cells.

### Hypoxia stimulates exosomal MMP-13 expression in HIF-1α-dependent manner

**Fig. 2:**
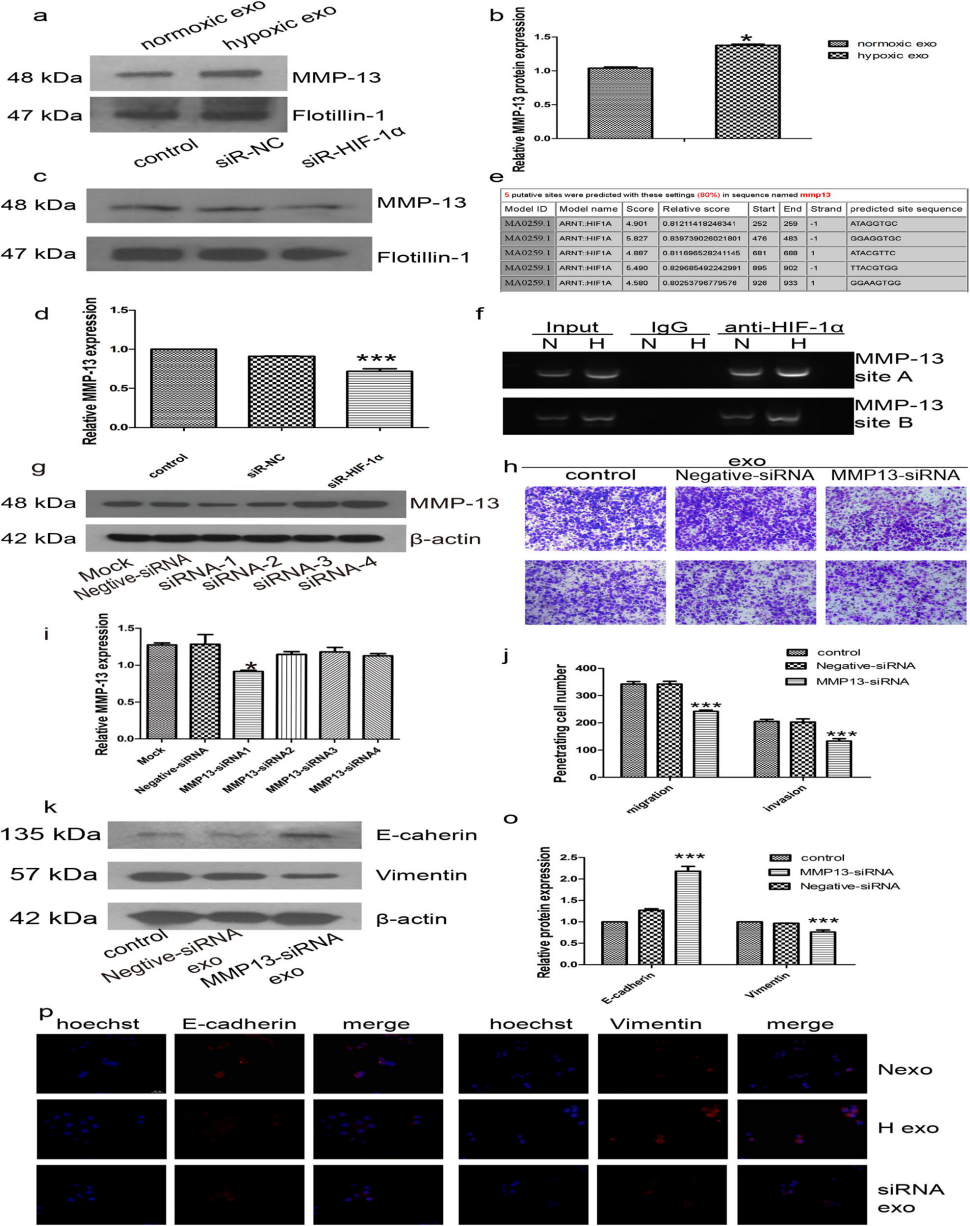
Hypoxia stimulates exosomal MMP-13 expression in an HIF-1α-dependent manner that facilitates cell migration and invasion. **a**, **b** Western blot analysis of MMP-13 levels in normoxic or hypoxic exosomes. Flotillin-1 was used as a loading control. **c**, **d** After treatment with HIF-1α siRNA, the level of MMP-13 in exosomes purified from hypoxic CNE2 cells was analyzed. **e** Five sequences matching the consensus 5’-RCGTG-3’ sequences and located in a DNase I hypersensitive region of chromatin were tested for HIF-1α binding. **f** Chromatin of hypoxic CNE2 was immunoprecipitated with IgG or antibody against HIF-1α. PCR was performed with primers flanking a candidate HIF binding site in the MMP-13 gene. **g**, **i** The most effective MMP-13-siRNAs under hypoxia were selected by western blot. **h**, **j** Exosomes purified from MMP-13-siRNA-treated hypoxic CNE2 cells decreased the migration (upper) and invasion (lower) ability of hypoxic CNE2 cells. **k**–**o** Exosomes purified from hypoxic CNE2 cells with or without MMP-13-siRNA treatment, then co-cultured with CNE2 cells for 24 h. Western blot analyzed the EMT markers in different exosome-treated CNE2 cells. Hypoxic CNE2 cells were used as a control. **p** Immunofluorescence staining analysis of cytoplasmic expression of EMT markers in different exosome-treated CNE2 cells. Data represent at least three experiments performed in triplicate, * indicates 0.01 < *p* < 0.05; ***, *p* < 0.001

Exosomes have been demonstrated to contain protein and miRNAs, which can be delivered to other cells and affect cellular function^20^. Our previous study showed that exosomes purified from the NPC patients’ peripheral plasma contained MMP-13 and promoted NPC metastasis. Hypoxia has been reported to enhance MMP activity; however, MMP activity in hypoxic NPC exosomes has not been studied. After oxygen deprivation for 24 h, the level of MMP-13 was up-regulated in hypoxic exosomes compared with exosomes purified under normal conditions (Fig. 2a, b) as well as in cells (Supplementary Figure 3a-c). Consistent with previous studies, the level of HIF-1α mRNA was unchanged even under hypoxic conditions, while the expression of MMP-13 was up-regulated (Supplementary Figure 3c). To further study whether hypoxia-induced MMP-13 expression was HIF-1α dependent, CNE2 cells were treated with the most effective HIF-1α-target small interfering RNA (siRNA; Supplementary Figure 3d). In normoxic conditions, cellular and exosomal MMP-13 levels were not significantly affected by HIF-1α knockdown (Supplementary Figure 3f-h). However, in hypoxic conditions, cellular (Supplementary Figure3i-l) and exosomal (Fig. 2c, d) MMP-13 levels were decreased by HIF-1α knockdown. These results suggested that hypoxia-induced cellular and exosomal MMP-13 expression is dependent on HIF-1α in CNE2 cells. To determine whether MMP-13 was a direct HIF-1α target gene, we analyzed the MMP-13 locus for the presence of consensus HIF binding site sequences (Fig. 2e). Chromatin immunoprecipitation (ChIP) assays were used to further validate the binding of HIF-1α to the predicted hypoxia-responsive element (HRE) region of MMP-13. PCR products corresponding to the MMP-13 HRE-containing promoter region were detected in cells cultured in 1% O_2_ after HIF-1α immunoprecipitation (Fig. 2f). These results confirmed the direct binding of HIF-1α to MMP-13 upon exposure to hypoxia.

### MMP-13 mediated hypoxic exosome-induced cell migration and invasion

Our previous study showed that NPC exosomes that were taken up by receipt cells in the tumor microenvironment resulted in higher EMT and angiogenesis^18^. To study the function of exosomal MMP-13, we treated hypoxic CNE2 cells with MMP-13 targeted siRNA (Fig. 2g, i), and siRNA-1 was selected for subsequent trials. CNE2 cells were treated with hypoxic exosomes derived from MMP-13 knockdown cells. As shown in Fig. 2h, j, the knockdown of MMP-13 exosomes significantly decreased the migration (upper) and invasion (lower) of target cells compared with hypoxic exosomes. Moreover, exosomal MMP-13 markedly decreased the E-cadherin level, which acts as an epithelial cell marker, while the level of the mesenchymal marker Vimentin was up-regulated (Fig. 2k, o, p) in exosome-treated cells. Various transcription factors such as Twist, Snail, Slug, ZEB1 and ZEB2 are known to orchestrate EMT by activating the crosstalk of signaling networks that confer traits of self-renewal and invasiveness to cancer cells. We further explored whether other factors in exosomes involved in EMT were aberrantly expressed under hypoxic conditions. As shown in Supplementary Figure 4, there was no significant difference in the expression of Slug and Snail in exosomes. The data showed that hypoxic exosome-induced cell migration and invasion was dependent on MMP-13 by mediating the progression of EMT of the target cells. Further study showed that hypoxic exosomes were taken up by receipt human umbilical vein endothelial cells (HUVECs; Supplementary Figure 5c), which facilitated the proliferation (Supplementary Figure 5a) and tube formation (Supplementary Figure 5e) of HUVECs. MMP-13 knockdown decreased the exosomal properties mentioned above (Supplementary Figure 5b, d, and e).

### Tumor growth was diminished by exosomes from MMP-13 knockdown cells

**Fig. 3:**
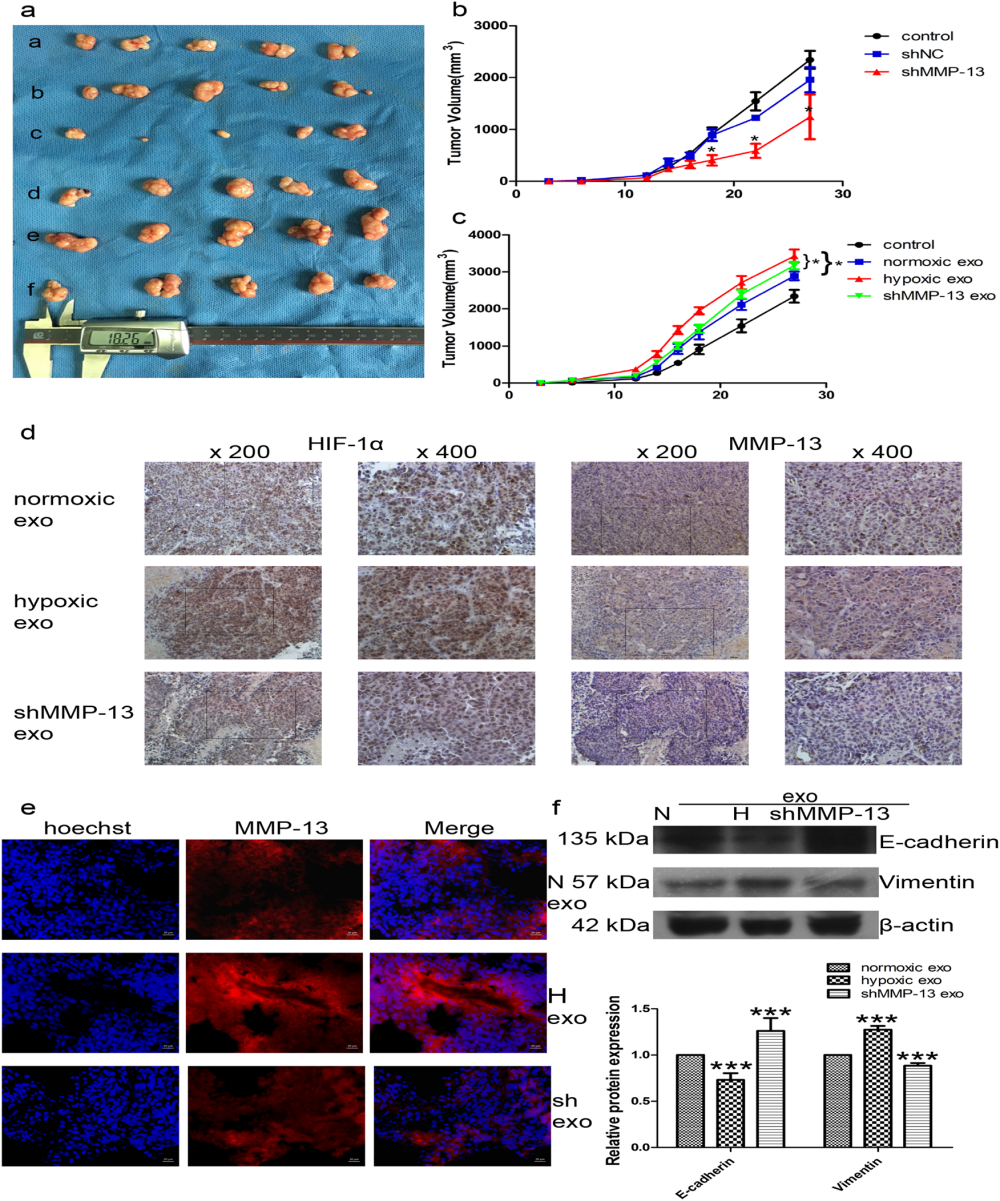
Tumor growth was diminished by exosomes from MMP-13 knockdown cells. **a** Tumorigenicity of CNE2 cells (a-a), scrambled shRNA treated cells (a-b), MMP-13 shRNA-treated cells (a-c), CNE2 cells with normoxic exosomes (a-d), CNE2 cells with hypoxic exosomes (a-e) and CNE2 cells with shMMP-13 exosomes (a-f). **b** Tumor growth of CNE2 cells with different levels of MMP-13 protein over time. **c** Tumor growth of NPC xenograft treated with different exosomes. **d** Immunohistochemistry detection of HIF-1α and MMP-13. Scale bar: 500 μm. **e** Immunofluorescent images of CNE2 cells stained for MMP-13 (red) and Hoechst (blue). Scale bar: 20 μm. **f** E-cadherin and Vimentin were measured by western blot; *n* = 5/group, tumor volume was periodically measured for each mouse and growth curves were plotted. Parametric generalized linear model with random effects. Data represent at least three experiments performed in triplicate, * indicates 0.01 < *p* < 0.05; ***, *p* < 0.001

To further confirm the effect of MMP-13, we performed an in vivo tumorigenesis experiment in nude mice. Tumor volumes and growth rates were significantly decreased in tumors derived from shMMP-13-treated CNE2 cells (Fig. 3a, b) (Supplementary Figure 6). As shown in Supplementary Figure 6, the expression of MMP-13 was reduced by MMP-13 small hairpin RNA (shRNA). These tumors also exhibited a reduction in MMP-13 expression by immunohistochemistry but unchanged HIF-1α expression (Supplementary Figure 7). Consistent with these results, MMP-13 exerted a significant inhibitory effect on tumor genesis in vivo.

Furthermore, to investigate the role of tumor-derived exosomal MMP-13 in tumor growth and metastasis, normoxic and hypoxic exosomes purified from MMP-13 shRNA-treated cells (200 μg/ml) were then injected into the xenograft tumors. The hypoxic exosomes increased the growth of the tumor. MMP-13 knockdown reversed the promotion of tumor growth by hypoxic exosomes (Fig. 3a, c). MMP-13 expression decreased in CNE2 tumors treated with shMMP-13 exosomes (Fig. 3d, e). We next investigated whether exosomal MMP-13 regulates EMT in vivo. Consistent with the in vitro experiments, hypoxic exosomes and MMP-13 knockdown exosome-treated tumors had decreased expression of E-cadherin as well as markedly induced the expression of Vimentin (Fig. 3f).

These data indicated that the hypoxic microenvironment could stimulate tumor cells to produce exosomes that carry greater amounts of MMP-13. These MMP-13-rich exosomes would then potentially induce non-hypoxic cells to invade and metastasize.

### HIF-1α and MMP-13 were over-expressed and associated with invasion and metastases in NPC tissues

**Fig. 4:**
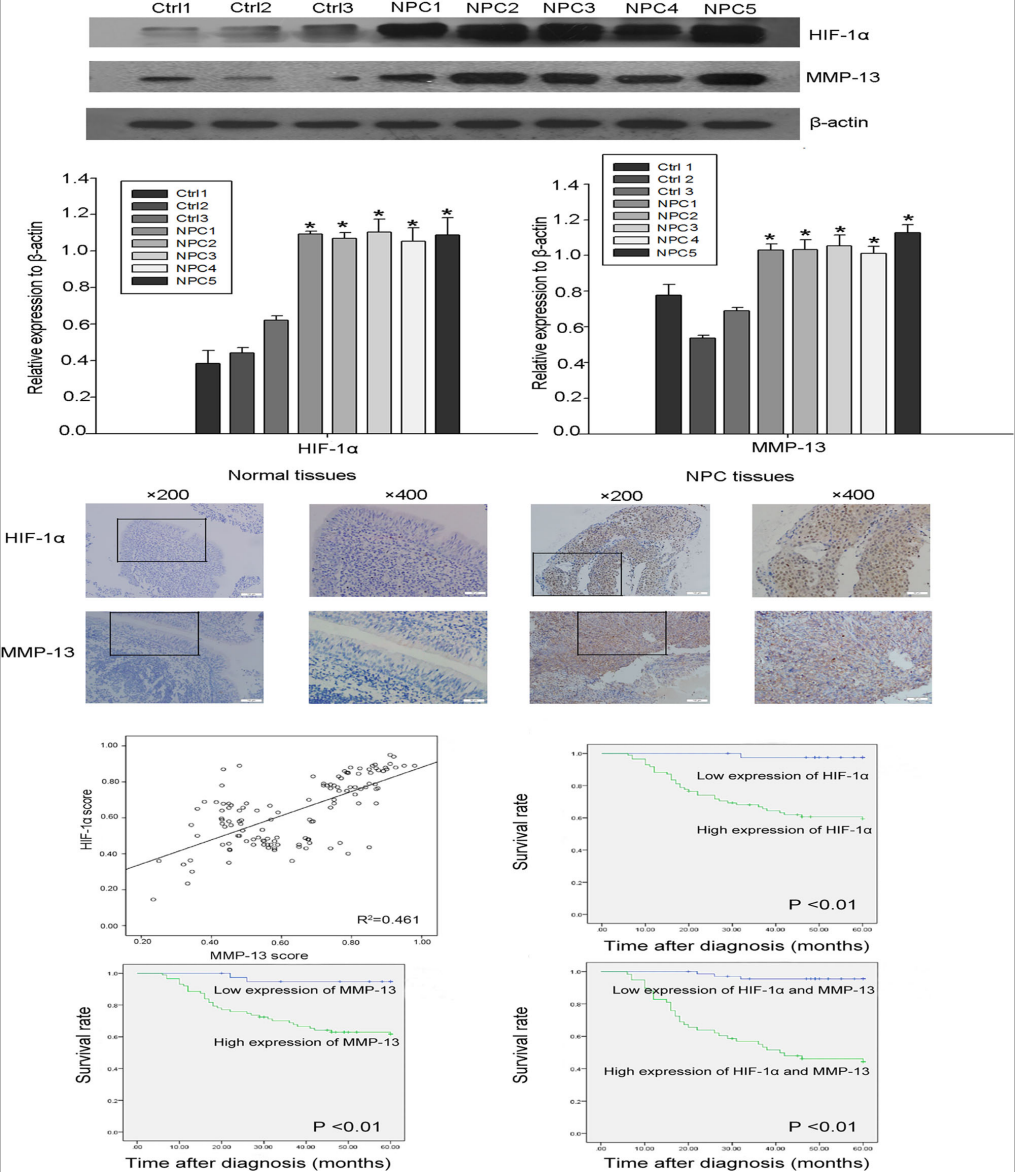
Relationship between expression of HIF-1α and MMP-13 proteins and clinicopathological features which predicts poor prognosis. **a**, **b** Expression levels of HIF-1α and MMP-13 in NPC tissues compared to the normal by western blot. **c** Immunohistochemistry results showed the protein level of HIF-1α and MMP-13 in NPC tissues. **d** Scatter plot of HIF-1α versus MMP-13 with regression line showing the correlation of them using Spearman’s correlation coefficient (*P* < 0.01). **e**–**g** Kaplan–Meier analysis of survival of patients with NPC expressing HIF-1α (**e**), MMP-13 (**f**) and both proteins (**g**) (*P < *0.001, log-rank test). Data represent at least three experiments performed in triplicate, * indicates 0.01 < *p* < 0.05

As our previous work confirmed that MMP-13 was over-expressed in exosomes purified from NPC patients, further work was done to analyze the correlation between MMP-13, HIF-1α and clinical–pathologic parameters. Western blot showed that in the fresh NPC tissues, HIF-1α and MMP-13 were over-expressed compared to normal tissue samples (Fig. 4a, b). To gain further insight into the prognostic value of HIF-1α and MMP-13 expression in patients with NPC, paraffin-embedded tissue sections (*n* = 126) were examined using immunohistochemistry (Fig. 4c). High HIF-1α expression was localized to the nuclei and cytoplasm in 69.05% (87/126) of the resected tumor tissue samples. MMP-13-positive staining was observed in the cytoplasm of NPC cells in 67.46% (85/129) of the NPC samples. No positive expression of HIF-1α was detected in 30 normal control samples, which was in accordance with the previous study^24^. A total of 23.3% (7/30) of sections had MMP-13-positive expression. The positive rates of HIF-1α and MMP-13 expressions in NPC were significantly higher than those in the normal controls (*P < *0.05). These results above suggested that high levels of HIF-1α and MMP-13 could be potential detections or prognostics biomarkers for NPC.

### Relationship between HIF-1α and MMP-13 protein expression and clinicopathological features that predict poor prognosis

Spearman's analysis further revealed that HIF-1α expression was positively correlated with MMP-13 expression (*r = *0.679, *P* < 0.01) in NPC (Fig. 4d). Immunohistochemical determination of HIF-1α and MMP-13 levels was also statistically analyzed to identify their association with the clinicopathologic features of NPC. HIF-1α expression was significantly correlated with lymph node stage (*P* = 0.031) and clinical stage (*P* = 0.001), but there was no significant association with gender, age and T stage (all *P* > 0.05). Moreover, high MMP-13 expression was significantly associated with T classification (*P* = 0.043), lymph node metastasis (*P* = 0.008) and clinical stage (*P* < 0.01) of patients with NPC. There was no relationship between MMP-13 expressions and age or gender (all *P* > 0.05) (Table 1). The determination of the prognosis of the NPC patients with HIF-1α expression level was also investigated in our work. Prior to the date of the last follow-up, our data showed that overall survival time was shorter in the HIF-1α-positive group compared with the HIF-1α-negative group (*P < *0.01). The same result was shown in MMP-13-positive cases (*P < *0.01). Kaplan–Meier survival curves also showed that patients with both HIF-1α- and MMP-13-positive expressions were associated with poorer overall survival than those with both HIF-1α- and MMP-13-negative expressions (*P < *0.01) (Fig. 4e–g). Univariate analyses showed that T classification (*P* = 0.034), lymph node metastases (*P* < 0.01), clinical stage (*P* < 0.01), HIF-1α expression (*P* < 0.01) and MMP-13 expression (*P* < 0.01) were all significantly correlated with patients’ survival (Table 2). These data suggest that HIF-1α is correlated with MMP-13 expression in NPC samples and can predict poor prognosis of NPC patients before treatment.

**Table 1:** Relationship between HIF-1α, MMP-13 expression and clinicopathological parameters in 126 NPC specimens

Clinicopathological parameters	Total	HIF-1α		*P*-value	MMP-13		*P*-value
		Low	High		Low	High	
Gender							
Male	97	31	66	0.655	31	66	0.799
Female	29	8	21		10	19	
Age (years)							
<50	35	15	20	0.073	14	21	0.268
≥50	91	24	67		27	64	
T classification							
T1 + 2	73	24	49	0.583	29	44	0.043*
T3 + 4	53	15	38		12	41	
N classification							
N0	12	7	5	0.031*	8	4	0.008*
N1–3	114	32	82		33	81	
Clinical stage							
I–II	29	16	13	0.001*	17	12	0.001*
III–IV	97	23	74		24	73	

*Statistical analyses were performed by the Pearson χ^2^ test. *P* < 0.05 was considered significant

**Table 2:** Survival status and clinicopathological parameters in 126 human nasopharyngeal carcinoma tissues

Clinicopathological parameters	No.	Survival status, *n* (%)		*P*
		Alive	Dead	
Gender				
Male	97	69	28	0.614
Female	29	22	7	
Age (year)				
<50	35	26	9	0.747
≥50	91	65	26	
T classification				
T1–T2	73	58	15	0.034^*^
T3–T4	53	33	20	
N classification				
N0	12	12	0	<0.01^*^
N1–3	114	79	35	
Clinical stage				
I–II	29	28	1	<0.01^*^
III–IV	97	63	34	
HIF-1α				
Low expression	39	37	2	<0.01^*^
High expression	87	54	33	
MMP-13				
Low expression	41	40	1	<0.01^*^
High expression	85	51	34	

*Statistical analyses were performed by the Pearson χ^2^ test. *P* < 0.05 was considered significant

In summary, our data offer convincing evidence that the hypoxic microenvironment stimulates NPC cells to produce exosomes containing greater amounts of MMP-13. These MMP-13-rich exosomes could participate in the progression of EMT to convert normoxic cells to a malignant phenotype.

## Discussion

NPC is a highly invasive malignancy with early cervical lymph node metastases at the time of diagnosis. Hypoxia is an important element of the tumor microenvironment^25,26^, and is associated with aggressive tumor phenotypes and poor patient outcomes^27^. A previous study showed that all primary NPC tissues contain hypoxic regions which were associated with increased distant metastases, decreased local control and resistance to chemotherapy in advanced NPC patients^4^. HIF-1α is a central component of hypoxia that binds to hypoxic-responsive elements and up-regulates hypoxic-regulated genes, inducing changes that enable tumor cells to survive apoptosis, angiogenesis and metastases^28^. Furthermore, under hypoxic conditions, HIF-1α rapidly accumulates and trans-activates hundreds of genes, such as MMPs^29^. In our study, we found that HIF-1α and MMP-13 were over-expressed in NPC tissues. To gain further insight into the prognostic value of the biologic and clinicopathologic significance of HIF-1α and MMP-13 in NPC, immunohistochemical staining analysis showed that MMP-13 and HIF-1α over-expression were correlated with the lymph node metastases, clinical stage and poor prognosis of patients with NPC (*P* < 0.05), but not with age, gender and T stage. HIF-1α expression was positively correlated with MMP-13 (*r = *0.679, *P < *0.001). These data suggested MMP-13 had an oncogenic role in NPC as well as HIF-1α.

Exosomes are nano-sized (50–100 nm in diameter) membrane-bound vesicles that contain a wide range of functional proteins, mRNAs, and miRNAs^30^. Ohyashiki et al. had demonstrated that exosomal miR-135b shed from hypoxic multiple myeloma cells enhances angiogenesis by targeting factor-inhibiting HIF-1^23^. In our study, the results of transwell and zebrafish tumor models showed that hypoxic NPC cell-derived exosomes enhanced the invasiveness and motility of naive NPC cells.

MMPs are a group of zinc-dependent proteolytic enzymes that stimulate tumorigenesis and establish metastatic foci at the secondary sites^27^. MMP-13 is an important type of MMPs that is often over-expressed in various tumors and participates in tumor metastases. We provided evidence that hypoxia increased the MMP-13 levels in tumor-derived exosomes in a HIF-1α-dependent manner. ChIP assay was further used to confirm that MMP-13 was a direct HIF-1α target gene.

Previously, HIF-1α, a major mediator of hypoxia, has been reported in some malignant tumors to promote EMT^24,31,32^. Our data showed that under oxygen deprivation conditions or treatment with CoCl_2_, HIF-1α was up-regulated in NPC cells, and the level increased more in the poorly differentiated CNE2 cells compared to the well differentiated CNE1 cells. As hypoxic exosomes enhanced the invasiveness and motility of normoxic NPC cells, hypoxia significantly increased the metastatic ability of CNE2 cells. Moreover, EMT occurred in hypoxic CNE2 cells regardless of cell morphology or the change of markers such as E-cadherin and Vimentin. Hypoxic CNE2 also showed increased metastatic ability compared to normal controls. Our data further demonstrated that hypoxic exosome-induced cell migration and invasion were dependent on MMP-13, which induced EMT of the recipient normoxic cell.

In conclusion, we provided evidence that hypoxia increased the MMP-13 levels in tumor-derived exosomes in a HIF-1α-dependent manner. Tumor-derived exosomes could function as a messenger that delivers MMP-13 between normoxic and hypoxic cancer cells and thus remodels the tumor microenvironment of NPC.

## Materials and methods

### Immunohistochemistry

All NPC specimens and normal nasopharyngeal epithelium samples were obtained from the Affiliated Hospital of Nantong University. No patients had received any anti-tumor treatments before biopsy. The procedure of human sample collection was approved by the Ethical Committee of the Affiliated Hospital of Nantong University. Informed consent was obtained from all patients.

Immunohistochemistry was used to analyze the expression of HIF-1α and MMP-13 in NPC. The procedure was as mentioned previously^33^. The slides were incubated with anti-human monoclonal antibody (Abcam, USA, 1:100), anti-human MMP-13 (Santa Cruz, CA, USA, 1:100). Briefly, according to the intensities of the nuclear, cytoplasm and/or membrane staining, staining intensity was scored as follows: 1, strong and moderate staining; 0, weak and no staining. Scores for the percentage of immunopositive cells were rated as follows: 0, <10% positive cells; 1, 10–25%; 2, 25–50%; and 3, >50% positive cells. Therefore, the combined staining scores (extent×intensity) could range from 0 to 3, and the cases were classified into negative group with scores ≦1 and positive group with scores >1.

### Cell culture and treatment

Human NPC cell lines CNE1 and CNE2 were as gift from the Sun Yat-Sen University and Xiang-Ya School of Medicine, which were tested by the standard SRT method. They were cultured in RPMI-1640 medium (Gibco, Grand Island, NY, USA) supplemented with 10% fetal bovine serum (Gibco BRL, Grand Island, NY, USA). The cells were kept in a humidified atmosphere containing 5% CO_2_ and 21%O_2_. HUVECs were purchased from ScienCell Research Laboratories which were cultured in Dulbecco's modified Eagle's medium low glucose (HyClone, Logan, UT, USA).

#### Hypoxia treatment

Cells were incubated in a sealed incubator chamber that provided 1% O_2_, 5% CO_2_ and 94% N_2_ (Billups Rothenberg). Hypoxia was also induced by exposing cells to CoCl_2_ (Sigma‑Aldrich, St. Louis, MO, USA) in serum-free medium under normal conditions.

### Exosome isolation and purification

Exosomes were isolated from CM as previously described^18^. The isolation method included a penultimate centrifugation step to remove small cell debris and then ultracentrifugation at 100,000 ×* g* for 1 h to generate an exosome pellet (Type 90 Ti rotor; Beckman Coulter, Fullerton, CA). The pellets were then washed once with phosphate-buffered saline (PBS).

### Electron microscopy

Purified exosome pellets were fixed with 2.5% glutaraldehyde and then centrifuged at 100,000 × *g* to remove the glutaraldehyde. The pellets were then negatively stained by 3% aqueous phosphotungstic acid and fixed on copper mesh formvar grids. Samples were observed using the JEOL Transmission Electron Microscope (JEM-1230; JEOL, Tokyo, Japan).

### Western blot assay

Equal amounts of proteins were separated on 10% sodium dodecyl sulfate–polyacrylamide gel electrophoresis gel and transferred to polyvinylidene difluoride membranes. After blocking, the membrane was incubated with primary antibody against MMP-13, β-actin (Santa Cruz Biotechnology, CA, USA), HIF-1α (Abcam, USA), Vimentin, E-cadherin, Slug and Snail (Cell Signaling Technology, Danvers, MA, USA). Horseradish peroxidase (Santa Cruz Biotechnology, CA, USA)-conjugated secondary antibody was used and then visualized with ECL reagents.

### Transwell migration and invasion assay

#### Migration

The 5 × 10^4^ CNE2 or HUVECs were plated into the upper chambers of cell culture inserts (24-well type, 8 µm pore size, Millipore, MA), which were placed in medium containing 10% fetal bovine serum with or without exosomes. After 16 or 20 h of incubation at 37 °C, the chamber was washed and cells inside the upper chamber were removed. Cells on the lower membrane surface were fixed and stained with 0.25% crystal violet, and counted for 5 random per well. Cell counts are expressed as the mean number of cells per field of view.

#### Invasion

Transwell chambers were coated with Matrigel (BD, Bedford, MA, USA) and incubated. Cells (3 × 10^5^) were plated into the top side of polycarbonate transwell filter and incubated for 24 h. Other processes were doing as migration.

### Quantitative real-time PCR (qRT-PCR) analysis

Total RNA was extracted using Trizol® reagent (Invitrogen) from cells. The qRT-PCR was performed according to the instructions for Power SYBR Green PCR Master Mix (Applied Biosystems, USA). The cycle was 30 min at 42 °C, 2 min at 94 °C followed by 35 cycles of 94 °C for 20 s, one cycle of 58 °C for 20 s and elongation at 72 °C for 30 s. The sequences of the primers were as follows: MMP-13 (forward: GTGGTGGTGATGAAGATG; reverse: TCTAAGGTGTTATCGTCAAG); HIF-1α (forward: ACTCAGGACACAGATTTAGACTTG; reverse: TGGCATTAGCAGTAGGTTCTTG) E-cadherin (forward: GCTGGACCGAGAGAGTTTCC; reverse: CGACGTTAGCCTCGTTCTCA) Vimentin (forward: AAATGGCTCGTCACCTTCGT; reverse: CAGCTTCCTGTAGGTGGCAA) and GAPDH served as the internal control. The experiment was performed in triplicate.

### siRNA transfection

The negative control siRNA (NC siRNA) and specific siRNAs were designed and obtained from Biomics Biotechnologies Co. Ltd (Nantong, China). HIF-1-α-targeted siRNA (sense, 5’-CGAUGGAAGCACUAGACAAdTdT-3’; antisense, 5’-UUGUCUAGUGCUUCCAUCGdTdT-3’) MMP-13-targeted siRNA (sense, 5’-GGAGAUAUGAUGAUACUAAdTdT-3’; antisense, 5’-UUAGUAUCAUCAUAUCUCCdTdT-3’) was chosen out of 4 individual siRNAs. Scrambled-sequence siRNA duplex was used as negative siRNA control.

### Cell viability assay

Cells were seeded into 96-well plates and assessed by CCK8 assay (Beyotime Institute of Biotechnology, Haimen, China). The absorbance of each well was read on a microplate reader (F-2500 Fluorescence Spectrophotometer; Hitachi) at 450 nm.

### Chromatin immunoprecipitation assay

ChIP assays were performed using a ChIP Assay Kit (Millipore) according to the manufacturer’s instructions. Briefly, cells were fixed, lysed and sonicated to obtain DNA fragments in arranging in size from 200 to1000 bp. Chromatin was then precipitated with nonspecific IgG antibodies (Millipore), anti-HIF-1α (Abcam). DNA was extracted and PCR was performed with primers for MMP-13 promoter fragment.

### Lentivirus production and infection

Lentiviral particles carrying LV-MMP-13-shRNA vector and their flanking control sequence (Mock for short) were constructed by GeneChem (Shanghai, China). CNE2 cells were infected with lentiviral vector and the expression of MMP-13 was confirmed by western blot.

### Animal xenograft tumor model

In vivo study was approved by the committee on the Ethics of Animal Experiments of Nantong University. All BALB/c at hymic nude mice (5 weeks old) were provided by Shanghai Laboratory Animal Center, China, and kept in a specific pathogen-free environment. All mouse experiments followed institutional guidelines. 1 × 10^6^ CNE2 cells transfected with shMMP-13 and scramble shRNA or the control (*N* = 5 per group) in 0.1 ml 1640 medium without fetal bovine serum were subcutaneously injected into the mice. Tumors were allowed to grow for 3 days and then injected 20 μg of total exosome protein (in a total volume of 100 μl of PBS) three times a week, and PBS was used as control. Tumors were measured with sliding calipers and volume was calculated as (length×width2)/2. After 27 days, the mice were killed and tumor tissues were fixed in 10% formalin overnight or kept at –80 °C for later research.

### Zebrafish tumor model

The development of zebrafish tumor model followed institutional guidelines^21^. Briefly, fertilized zebrafish eggs of the transgenic zebrafish *Tg (fli1a: GFP)* were incubated at 28 °C in an incubator. To prevent pigmentation, embryos at 22 h post fertilization (hpf) were treated with 0.2 mM 1-phenyl-2-thiourea. At 48 hpf, anesthetized zebrafish embryos were placed onto a modified agarose gel for microinjection. Before injection, 2 g/ml of DiI (Fluka, Germany) was used to label tumor cells in vitro. Approximately 100–500/5 nl tumor cells were resuspended in serum-free 1640 medium and then injected into the perivitelline cavity of each embryo using a microinjection system (WPI).

### Immunocytochemical analysis

Cells were grown on glass cover slips, washed and fixed with 4% paraformaldehyde. The cover slips were then incubated with 1% bovine serum albumin in PBS and with primary antibody overnight, washed and incubated with fluorescein isothiocyanate-labeled secondary antibodies (Earth Ox) and the nuclei were labeled with Hoechst (Invitrogen) for 1 h. The cover slips were then observed under an Olympus camera.

### Transmission electron microscopy

According to the manufacturer’s instructions, PKH67 (Sigma-Aldrich)-labeled exosomes were added to CNE2 cells. The cells were fixed and then processed as introduced immunocytochemical analysis. Images were collected with a TCS SP5 confocal microscope (Leica Microsystems, Wetzlar, Germany) as mentioned previously^18^.

### Statistical analysis

Each experiment was performed as least three times; data were presented as mean ± SEM. Statistical analyses were performed using SPSS17.0 software. A value of *P < *0.05 was considered statistically significant. Survival curves were estimated by Kaplan–Meier analysis and compared by the log-rank test. χ^2^ test was used to determine the significance of differences in multiple comparisons.

## Electronic supplementary material

Supplementary Fig. 1

Supplementary Fig. 2

Supplementary Fig. 3

Supplementary Fig. 4

Supplementary Fig. 5

Supplementary Fig. 6

Supplementary Fig. 7

Supplementary figure legends
