# Supplementary figures and images for: Hypoxia-Induced Matrix Metalloproteinase-13 Expression in Exosomes from Nasopharyngeal Carcinoma Enhances Metastases
Source: Cell Death Dis. 2018 Mar 7;9(3):382. doi: 10.1038/s41419-018-0425-0 (PMC5841433; doi:10.1038/s41419-018-0425-0)

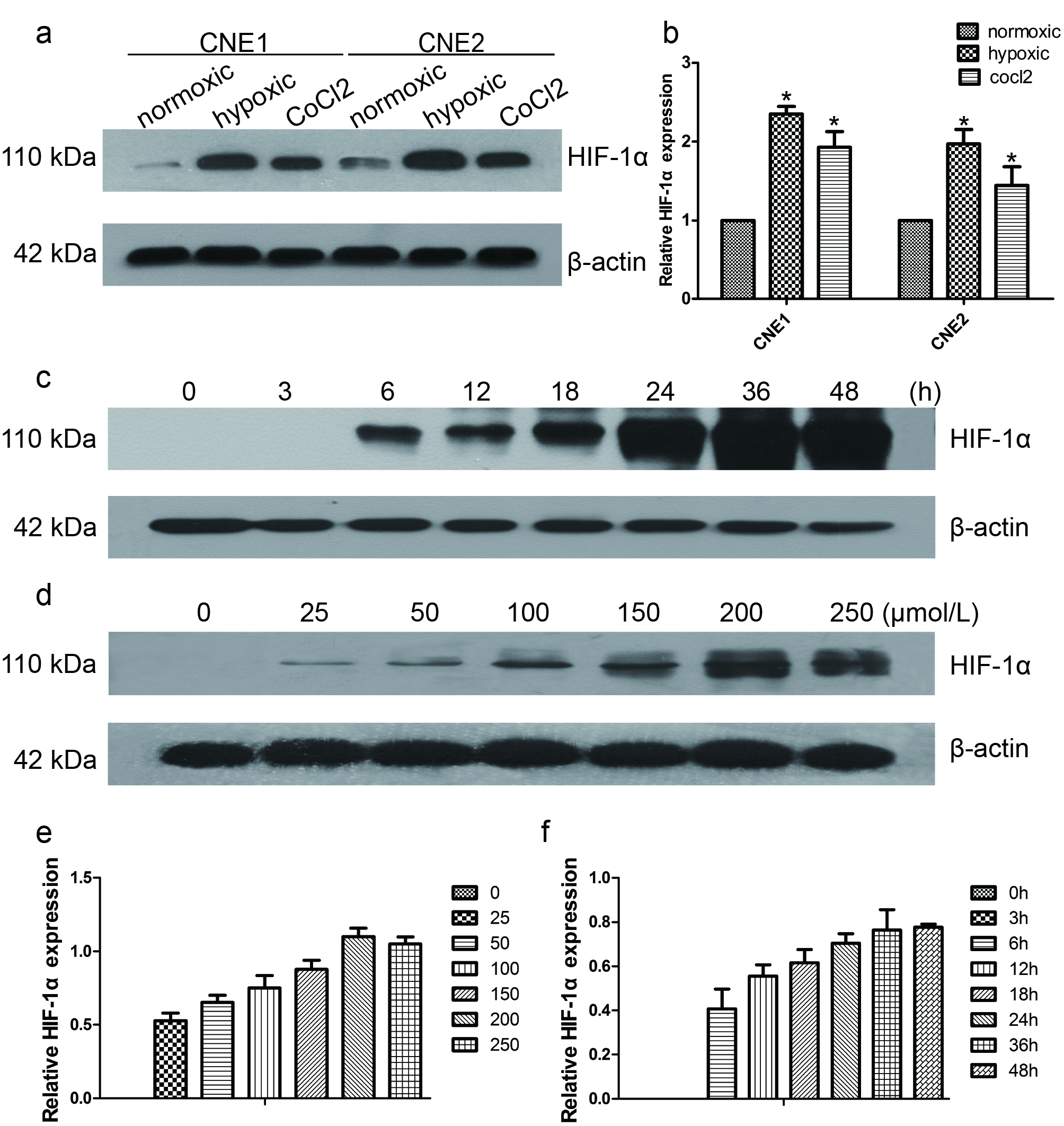

Supplement: Supplementary file 1 — Supplementary Fig. 1 [file 41419_2018_425_MOESM1_ESM.tif]

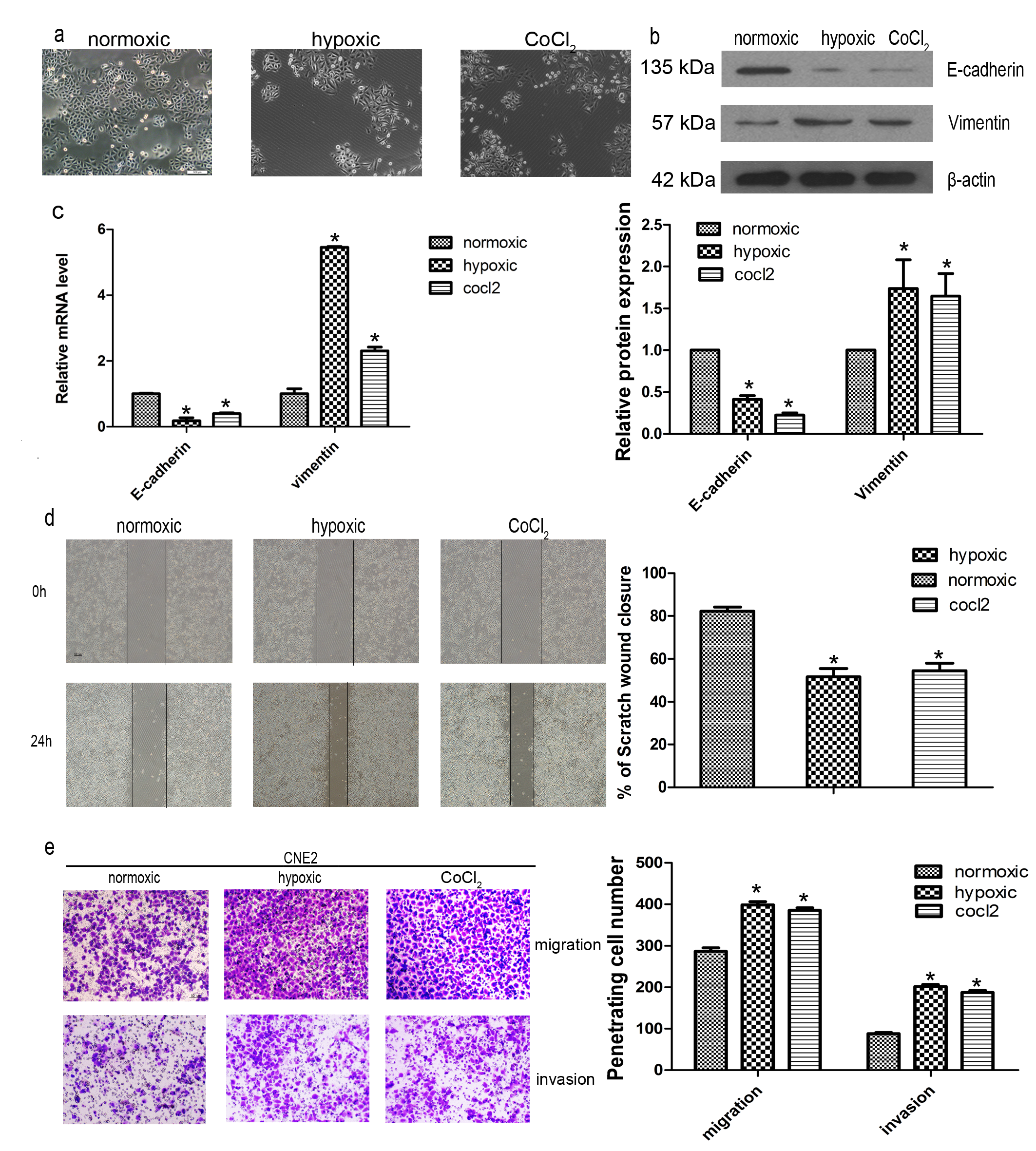

Supplement: Supplementary file 2 — Supplementary Fig. 2 [file 41419_2018_425_MOESM2_ESM.tif]

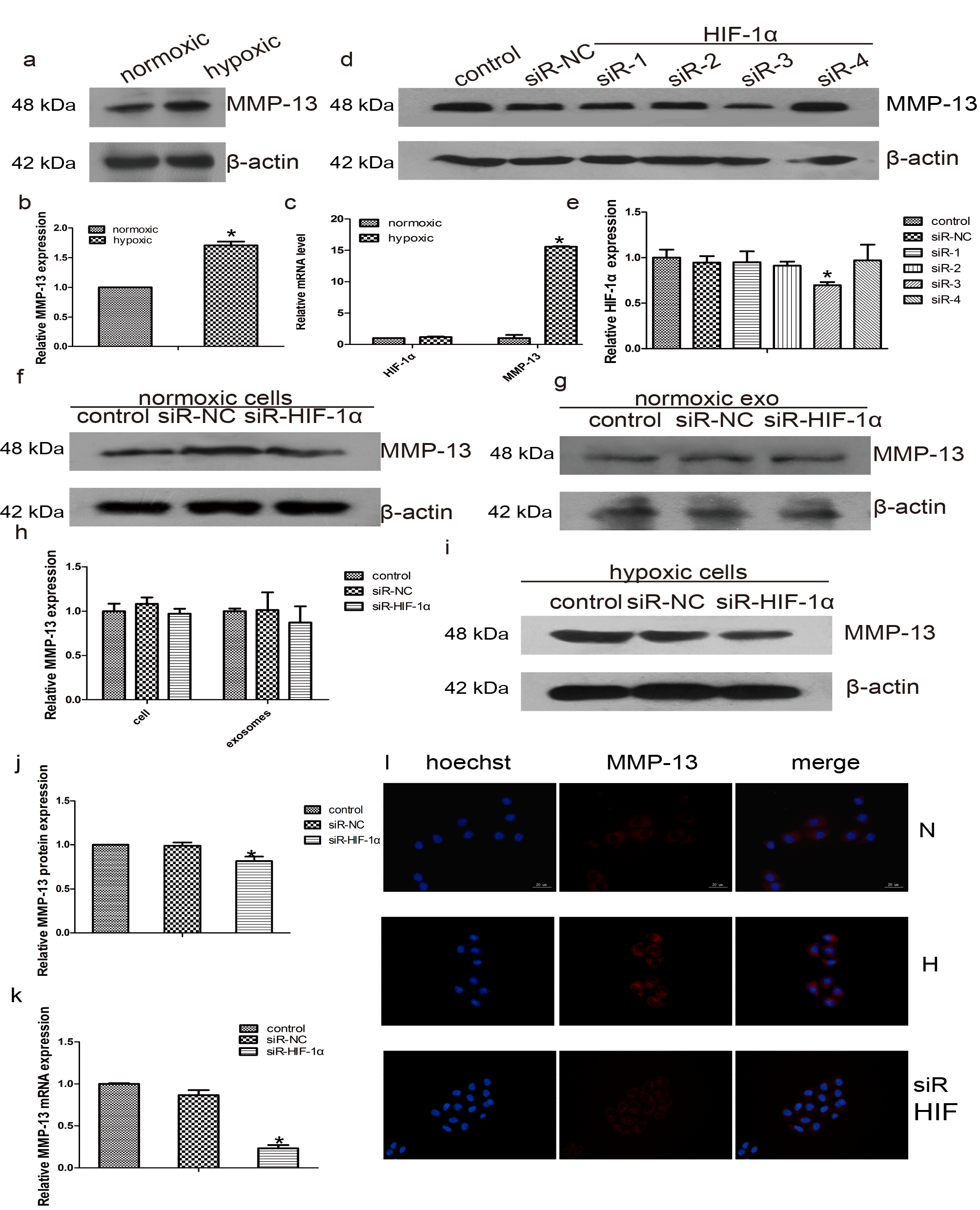

Supplement: Supplementary file 3 — Supplementary Fig. 3 [file 41419_2018_425_MOESM3_ESM.tif]

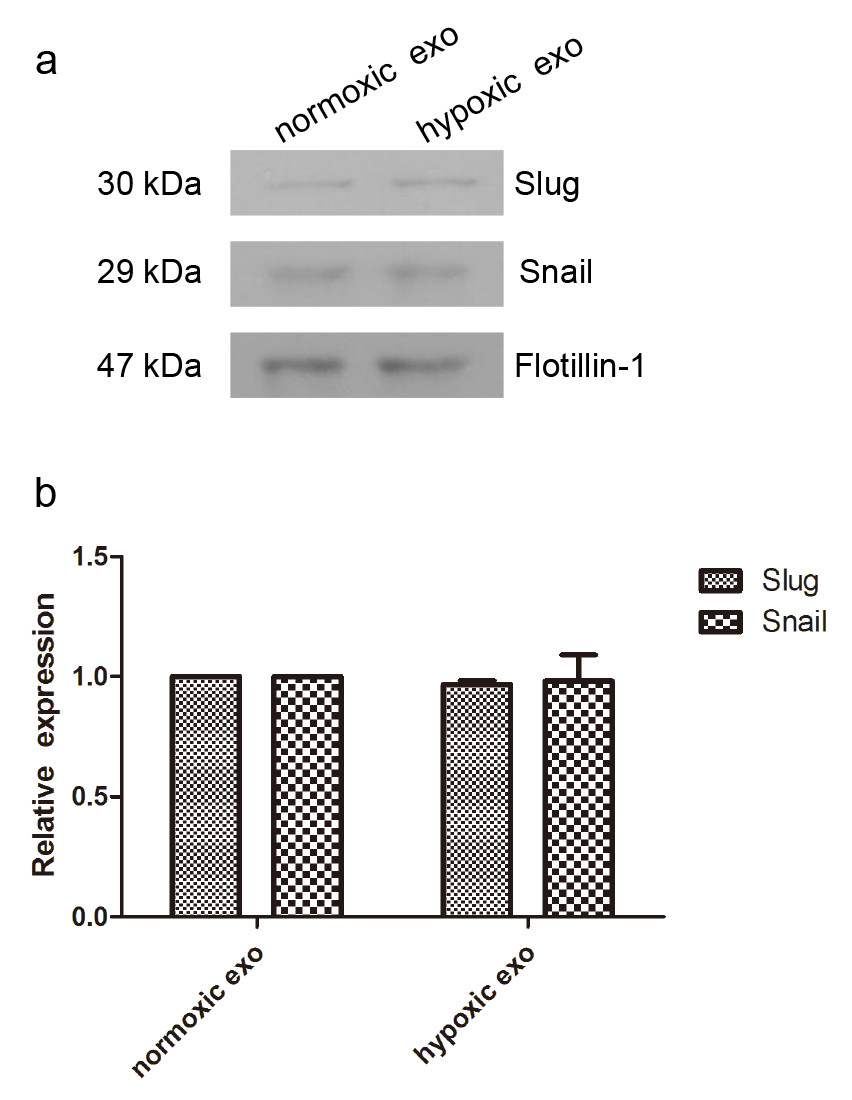

Supplement: Supplementary file 4 — Supplementary Fig. 4 [file 41419_2018_425_MOESM4_ESM.tif]

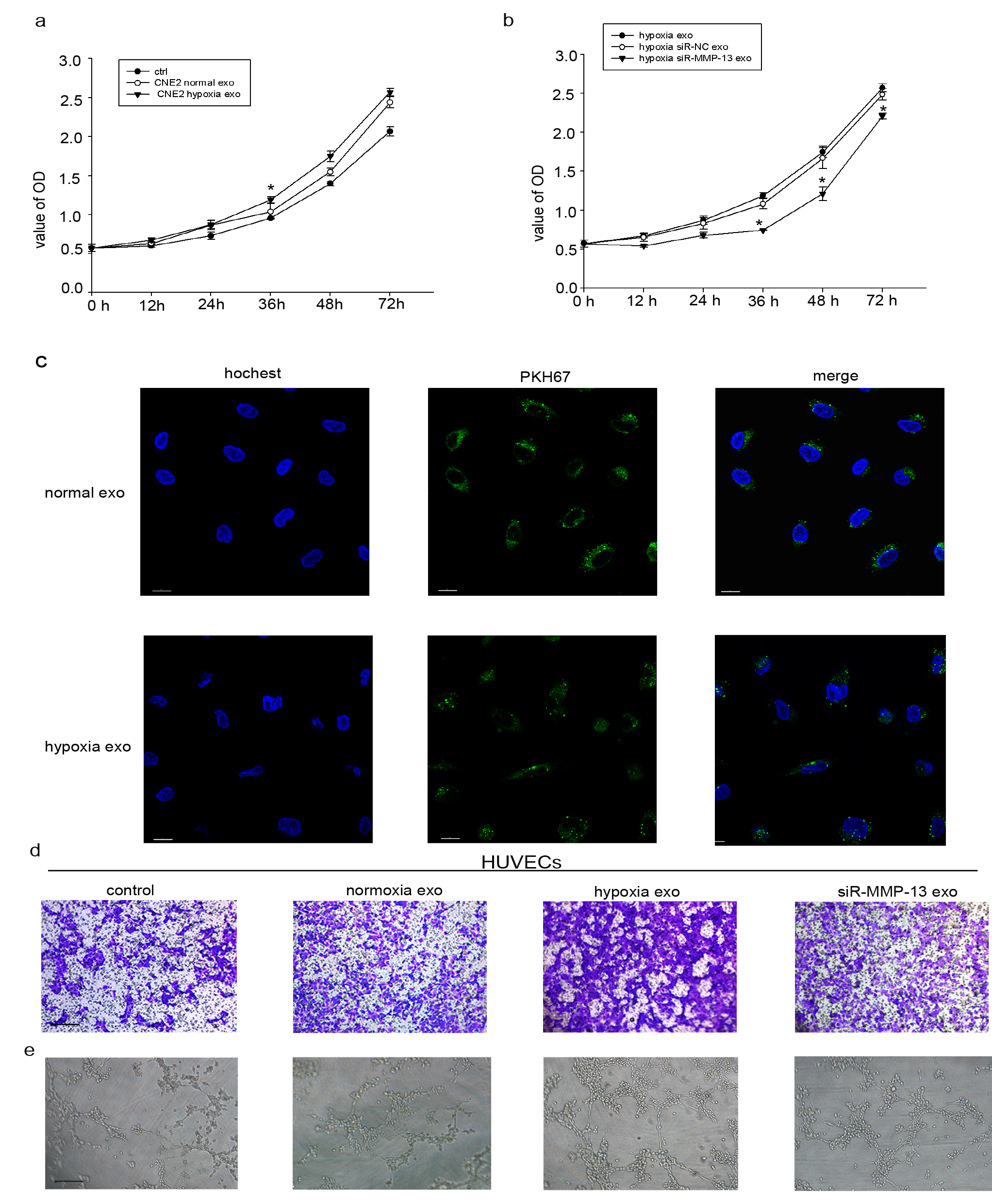

Supplement: Supplementary file 5 — Supplementary Fig. 5 [file 41419_2018_425_MOESM5_ESM.tif]

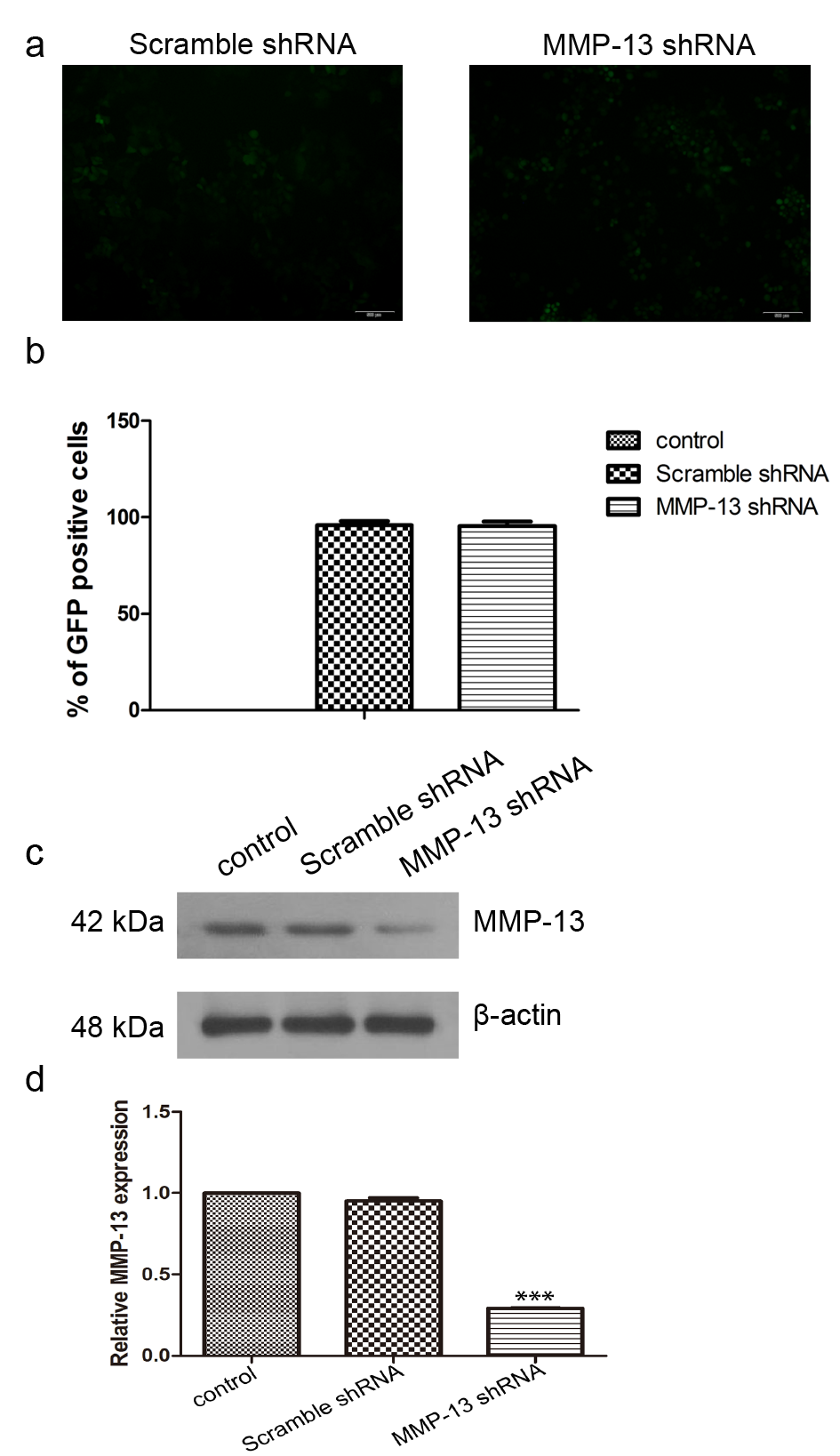

Supplement: Supplementary file 6 — Supplementary Fig. 6 [file 41419_2018_425_MOESM6_ESM.tif]

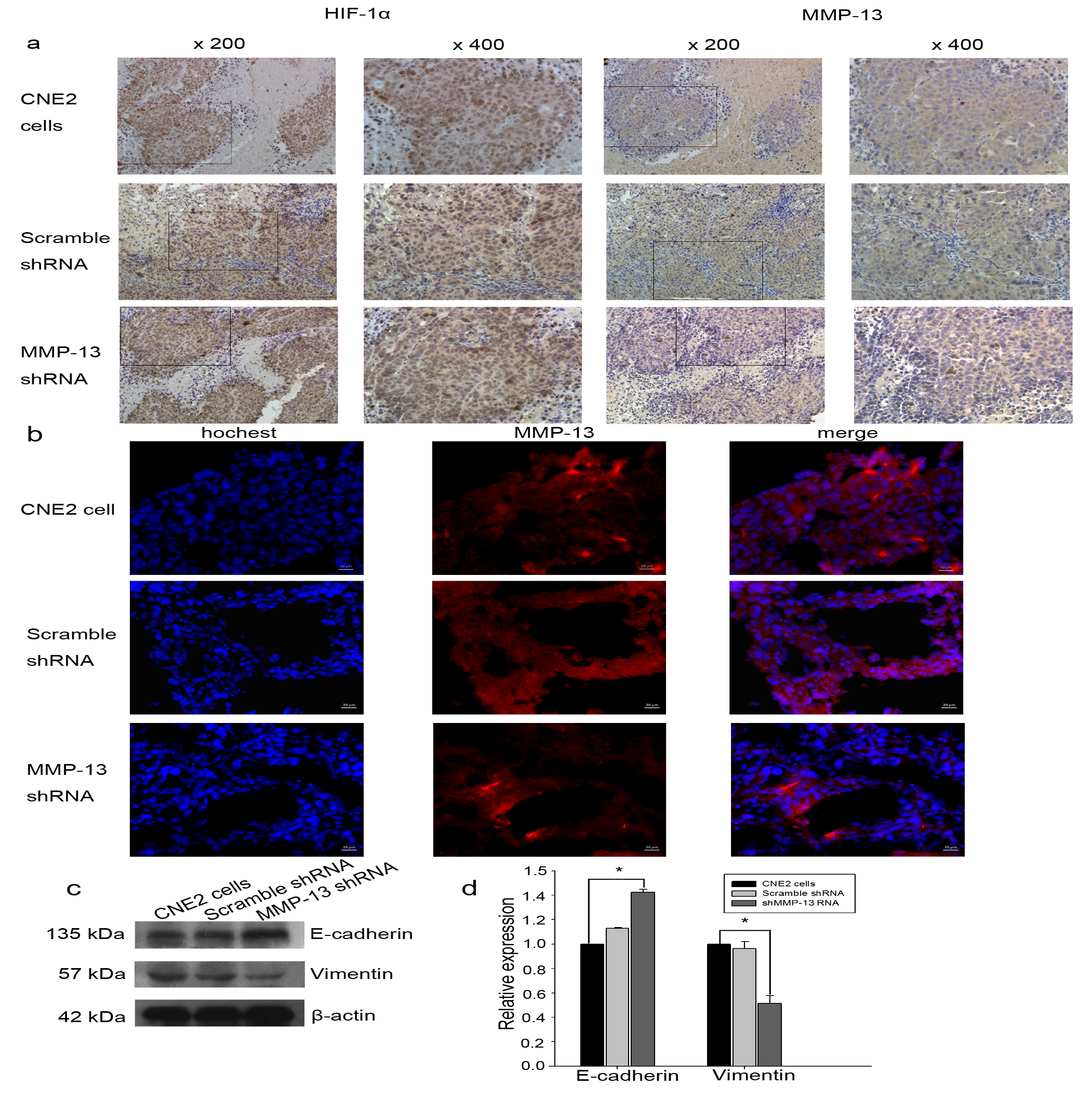

Supplement: Supplementary file 7 — Supplementary Fig. 7 [file 41419_2018_425_MOESM7_ESM.tif]
