# Supplementary material for: Hypoxia-Induced Matrix Metalloproteinase-13 Expression in Exosomes from Nasopharyngeal Carcinoma Enhances Metastases
Source: Cell Death Dis. 2018 Mar 7;9(3):382. doi: 10.1038/s41419-018-0425-0 (PMC5841433; doi:10.1038/s41419-018-0425-0)
Supplement: Supplementary file 8 — Supplementary figure legends [file 41419_2018_425_MOESM8_ESM.docx]

Supplementary figures legends

**Supplementary Figure 1 Hypoxia induced the over-expression of HIF-1**α **with time and dose dependence.**

(a-b) Western blot analysis of HIF-1α levels in the well differentiated NPC cell lines CNE1 and the poorly differentiated CNE2 cells under normal (normoxic), hypoxic conditions (hypoxic) or treated with CoCl_2_.

(c-f). HIF-1α level in CNE2 (c, e) [cultivate](javascript:void(0);)d under hypoxic conditions for 3-48 h; (d, f) treated with 0-250 μmol/L CoCl_2_.

**Supplementary Figure 2 Hypoxia induces EMT and enhances tumor migration as well as invasion.**

(a-c) CNE2 cells were maintained in either normoxia, hypoxia or CoCl_2_ and then analyzed by (b) Western blot and (c) qRT-PCR.

(d) Cells under hypoxic conditions (hypoxia, CoCl_2_) showed a faster wound closure rate than normal.

(e) Transwell analysis showed hypoxic conditions (hypoxia, CoCl_2_) significantly up-regulated the metastatic capacity of the CNE2 cells..

**Supplementary Figure 3 Hypoxia-induced HIF-1α over-expression facilitates MMP-13 expression.**

(a-b) Western blot and (c) qRT-PCR analysis of MMP-13 levels in CNE2 cells which were cultured under normoxic or hypoxic conditions. β-actin and GAPDH were used as loading control.

(d-e) Cells under hypoxia transfected with HIF-1α-siRNA and knockdown efficiency was detected by Western blot.

(f, g, h) Western blot analyzed cellular (f) and exosomal (g)MMP-13 levels, which were not significantly affected by either HIF-1α knockdown under normoxia.

(i-j) MMP-13 protein (i) and mRNA (j) levels were analyzed in cells by Western blot after transfection with the most effective HIF-1-α-siRNA under hypoxia.

(l) Immunofluorescence staining analysis of cytoplasmic expression of MMP-13 in HIF-1α-siRNA transfected hypoxic CNE2 cells. Red is MMP-13 staining. Blue is the nuclear staining by hoechst (Scale bar, 20 μm).

**Supplementary Figure 4 Analyzed other factors in exosomes involved in EMT.**

(a-b) Western blot analysis of Slug and Snail levels in exosomes that were purified from the CM of CNE2 cultured under normoxia or hypoxia. Flotillin-1 was used as a loading control.

**Supplementary Figure 5 MMP-13 mediated hypoxic exosomes induced HUVECs proliferation, migration and tube formation.**

(a) CCK8 assay showed hypoxic exosomes facilitated HUVECs proliferation.

(b) Exosomes purified from MMP-13-siRNA treated hypoxic CNE2 cells decreased the proliferation induced by hypoxic exosomes.

(c) Normoxic and hypoxic exosomes could be taken up by HUVECs.

(d) Transwell analysis of the effect of exosomes on HUVECs migration.

(e) Tube formation analyzed the HUVECs co-cultured with different exosomes.

**Supplementary Figure 6 Lentivirus were transfected to cells.**

(a) Representative microscopy image showing the cells were infected with lentivirus.

(b) Lentivirus treated GFP labeled cells were analyzed by flow cytometry.

(c-d) The MMP-13 shRNA-treated cells under hypoxia were analyzed by Western blot.

**Supplementary Figure 7 Decreased MMP-13 suppressed proliferation of NPC cells and participated in the progression in vivo.**

(a) Immunohistochemistry (IHC) detection of HIF-1α and MMP-13. Scale bar: 500 μm.

(b) Immunofluorescent images of CNE2 cells stained for MMP-13 (red) and hoechst (blue). Scale bar: 20 μm.

(c) E-cadherin and Vimentin were measured byWestern blot.

All experiments were carried out in triplicate and presented as the mean±SEM of three independent experiments. * indicates 0.01 < *p* < 0.05; ** indicates 0.001 < *p* < 0.01; *** indicates *p* < 0.001.
